# A network analysis of the interrelationships between depression, anxiety, insomnia and quality of life among fire service recruits

**DOI:** 10.3389/fpubh.2024.1348870

**Published:** 2024-07-03

**Authors:** Jian Liu, Zhen Gui, Pan Chen, Hong Cai, Yuan Feng, Tin-Ian Ho, Shu-Ying Rao, Zhaohui Su, Teris Cheung, Chee H. Ng, Gang Wang, Yu-Tao Xiang

**Affiliations:** ^1^Department of Rehabilitation Medicine, China Emergency General Hospital, Beijing, China; ^2^Unit of Psychiatry, Department of Public Health and Medicinal Administration, Institute of Translational Medicine, Faculty of Health Sciences, University of Macau, Macau, Macao SAR, China; ^3^Centre for Cognitive and Brain Sciences, University of Macau, Macau, Macao SAR, China; ^4^Unit of Medical Psychology and Behavior Medicine, School of Public Health, Guangxi Medical University, Nanning, China; ^5^Beijing Key Laboratory of Mental Disorders, National Clinical Research Center for Mental Disorders and National Center for Mental Disorders, Beijing Anding Hospital, Capital Medical University, Beijing, China; ^6^School of Public Health, Southeast University, Nanjing, China; ^7^School of Nursing, Hong Kong Polytechnic University, Kowloon, Hong Kong SAR, China; ^8^Department of Psychiatry, TheMelbourne Clinic and St Vincent’s Hospital, University of Melbourne, Richmond, Victoria, VIC, Australia

**Keywords:** depression, anxiety, quality of life, network analysis, insomnia

## Abstract

**Background:**

Research on the mental health and quality of life (hereafter QOL) among fire service recruits after the end of the COVID-19 restrictions is lacking. This study explored the network structure of depression, anxiety and insomnia, and their interconnections with QOL among fire service recruits in the post-COVID-19 era.

**Methods:**

This cross-sectional study used a consecutive sampling of fire service recruits across China. We measured the severity of depression, anxiety and insomnia symptoms, and overall QOL using the nine-item Patient Health Questionnaire (PHQ-9), seven-item Generalized Anxiety Disorder scale (GAD-7), Insomnia Severity Index (ISI) questionnaire, and World Health Organization Quality of Life-brief version (WHOQOL-BREF), respectively. We estimated the most central symptoms using the centrality index of expected influence (EI), and the symptoms connecting depression, anxiety and insomnia symptoms using bridge EI.

**Results:**

In total, 1,560 fire service recruits participated in the study. The prevalence of depression (PHQ-9 ≥ 5) was 15.2% (95% CI: 13.5–17.1%), while the prevalence of anxiety (GAD-7 ≥ 5) was 11.2% (95% CI: 9.6–12.8%). GAD4 (“Trouble relaxing”) had the highest EI in the whole network model, followed by ISI5 (“Interference with daytime functioning”) and GAD6 (“Irritability”). In contrast, PHQ4 (“Fatigue”) had the highest bridge EI values in the network, followed by GAD4 (“Trouble relaxing”) and ISI5 (“Interference with daytime functioning”). Additionally, ISI4 “Sleep dissatisfaction” (average edge weight = −1.335), which was the central symptom with the highest intensity value, had the strongest negative correlation with QOL.

**Conclusion:**

Depression and anxiety were important mental health issues to address among fire service recruits in the post-COVID-19 era in China. Targeting central and bridge symptoms identified in network analysis could help address depression and anxiety among fire service recruits in the post-COVID-19 era.

## 1 Introduction

Fire service recruits are a unique population affected by mental health issues. According to the regulations and policies in China, it is mandatory for fire service recruits to receive strict training for several months, pass stringent physical and professional assessment, and meet the full accreditation requirements before they can qualified and work as firefighters (1). As such, facing the pressure of meeting the high standards of assessments could increase the risk of mental health problems such as depression, anxiety and sleep problems among the fire service recruits.

The Coronavirus Disease 2019 (COVID-19) has resulted in a massive burden on the medical systems, as well as unprecedented health challenges for China and the global community (2). During the COVID-19 pandemic, the imposition of strict confinement measures and social isolation have also led to heightened psychological stress, significantly elevating the risk of mental health problems, such as depression, anxiety and insomnia (3, 4), in many subpopulations including fire service recruits. Apart from enduring extensive physical training and strict isolation measures during the COVID-19 epidemic, fire service recruits also experience prolonged detachment from the outside world, and separation from their relatives and friends. Consequently, the lack of social and familial support further increases the risk of mental health problems like anxiety, depression, and insomnia. Previous studies found that individuals who experienced strict isolation during the COVID-19 pandemic had a higher likelihood of adverse psychological outcomes, including depression, anxiety and insomnia, compared to those who had not experienced such isolation (5, 6). Moreover, studies conducted across various populations also found detrimental impact of the COVID-19 pandemic and associated lockdown measures on both physical and mental well-being of isolated individuals, which affected their quality of life (QOL) (7–9). However, there is a dearth of research data concerning the mental health of fire service recruits and its relationship to QOL in the post-COVID-19 era, thereby prompting further investigation into this population.

In recent years, network analysis has been increasingly employed in the field of mental health to better understand the interconnectedness between different psychiatric symptoms from the perspective of network (10, 11). The network graph visualizes the structure of the correlation matrix, which reflects the magnitude of correlation between symptoms, composing of core components referred to as “nodes” and “edges” (12, 13). Furthermore, by identifying highly connected central nodes, network analysis can provide insights into the risks, prevention, and interventions associated with specific syndromes (14). Further, network analysis can help identify psychiatric comorbidities that are important for prevention and treatment, as well as symptoms that serve as “bridge symptoms” for inter-disease transmission risk (15).

Network analysis has been widely applied across various psychiatric conditions such as anxiety, depression, insomnia, and QOL during the COVID-19 pandemic (16–20). For instance, a network analysis study on anxiety-depression-insomnia in Macao residents showed that “Sleep maintenance,” “Trouble relaxing” and “Interference with daytime functioning” were the most central (influential) symptoms during the COVID-19 pandemic (21). In the late stage of the COVID-19 pandemic, a network analysis study of anxiety-depression in Chinese nurses showed that “restlessness,” and “trouble relaxing” were the most central symptoms (22). The variation in the results between studies may be due to the differences in demographic features, methodology (e.g., sampling method and study design), workplace demands and environments, and the pandemic stage (23, 24).

There has been little research on fire service recruits, although some studies have examined the mental health status of firefighters in China. For instance, a study on mental health status of Chinese firefighters reported that cognitive reappraisal could negatively predict anxiety and depression symptoms (25). Another study found that mental health problems among firefighters in China were not uncommon and deserve more attention (26). Although fire service recruits and fire service recruits face stricter quarantine measures than the general population during and after the COVID-19 pandemic, and they also endure physically demanding training that can result in substantial physical fatigue and psychological stress (27, 28), there is insufficient research using network analysis to investigate psychiatric symptoms among fire service recruits in the post-COVID-19 era.

To address this gap, we employed network analysis to examine the interrelationships between depression, anxiety, and insomnia symptoms among Chinese fire service recruits in the post-COVID-19 era, with a focus on identifying central symptoms and bridge symptoms. Additionally, the study aimed to identify symptoms associated with depression, anxiety, and insomnia that impact on the QOL of Chinese fire service recruits, thus providing a theoretical foundation for targeted preventive interventions to address such symptoms and enhance their QOL.

## 2 Methods

### 2.1 Participants and study procedure

A consecutive sampling method was used in this multicentre, cross-sectional study that was conducted between February 13 and February 16, 2023. Due to the COVID-19 pandemic, face-to-face assessment could not be adopted; instead, an online survey was conducted. Following previous studies (29, 30), the online assessment was conducted on WeChat based on the “Questionnaire Star.” As all fire service recruits in the training centers had to report their health status regularly using WeChat during the study period, all participants were WeChat users.

All fire service recruits who received training across the four fire training centers located in Beijing, Sichuan, Guangxi and Guizhou during the study period were consecutively invited to participate on a voluntary and anonymous basis. A quick response code (QR code) was linked to the study invitation and questionnaire. Inclusion criteria were as follows: (1) fire service recruits aged 18 years or older; (2) were able to read Chinese and understand the purpose and content of the assessment. Based on relevant regulations in China, fire service recruits who were receiving training were not qualified to participate in firefighting mission tasks. Those with significant physical diseases and/psychiatric disorders (including COVID-19 complications and sequelae) were unable to receive regular training, therefore, could not be involved in this study. The study was approved by the ethics committee of the Emergency General Hospital and all participants signed the electronic written informed consent.

### 2.2 Measures

Following previous research (31, 32), the severity of depressive symptoms was measured using the validated Chinese version of the nine-item Patient Health Questionnaire (PHQ-9), which has good reliability, validity and has been widely used as a measure of depressive symptoms in various populations (33–35). The PHQ-9 consists of 9 items, with each scoring from 0 to 3 (from not at all to nearly every day) and the total score ranging from 0 to 27. A higher total score indicates more severe depressive symptoms, with a total score of 5, 10, 15, and 20, represent mild, moderate, moderate-to-severe and severe depressive symptoms, respectively (36).

Following previous research (37, 38), the severity of anxiety symptoms was measured using the validated Chinese version of the seven-item Generalized Anxiety Disorder scale (GAD-7) scale, which has satisfactory psychometric properties and has been widely used in various populations (39, 40). The GAD comprises seven items, each scoring from 0 (“not at all”) to 3 (“nearly every day”) (39, 41). The GAD-7 total score ranges from 0 to 21, with a higher total score indicating the more severe anxiety symptoms. The total scores of 5, 10, and 15 represent mild, moderate, and severe anxiety symptoms, respectively (42).

The severity of insomnia symptoms was measured using part of the Insomnia Severity Index (ISI) questionnaire (i.e., the items 1, 2, 3, 4, 5, and 6) (43–45), with each item ranging from 0 (none) to 4 (very severe). According to previous studies (46, 47), the PHQ-9, GAD-7 and ISI scores in this study were positively skewed. Therefore, we binarized each item: for all items, with a value of 0 was considered “absence” and recorded as 0; for PHQ-9 and GAD-7 items, each item with a value of 1 or 2 or 3 was considered “presence” and recorded as 1; and for ISI items, each item with a value of 1 or 2 or 3 or 4 was considered “presence” and recorded as 1 (22, 46, 47). Following previous research (48), overall QOL was measured using the first item of the World Health Organization Quality of Life-brief version (WHOQOL-BREF): “How would you rate your quality of life?,” with each item scored from 1 (extremely dissatisfied) to 5 (extremely satisfied) (49). In this network analysis, responses 1, 2 and 3 were recoded as 0, while responses 4 and 5 were recoded as 1 (50). Cronbach’s alpha values for the PHQ-9, GAD-7, ISI and the two-item WHOQOL were 0.858, 0.945, 0.896 and 0.846, respectively in the present study sample.

### 2.3 Data analysis

#### 2.3.1 Network estimation

The network analysis of depression, anxiety and insomnia model was performed using R software (51). This study applied the Ising model, which has been widely used in anxiety and/or depressive symptom networks conducted in different populations, to estimate the anxiety-depression-insomnia network (22, 47, 52). In this study, the score distributions of PHQ-9, GAD-7 and ISI items were all skewed. Therefore, following previous research (53–55), the Ising model was used to estimate this network. In the Ising model, all PHQ-9, GAD-7, and ISI items were divided into two categories, with “0” and “1” representing the absence and presence of depression, anxiety, or insomnia symptoms, respectively. Scores of “0” for all items were considered to indicate the absence of depression, anxiety, or insomnia symptoms, while scores of “1,” “2,” and “3” were considered to reflect the presence of depression, anxiety, or insomnia symptoms (56). The Ising model has also been widely used in the construction of network models for psychological binary data (53). In the Ising network, each item of the PHQ-9, GAD-7 and ISI is viewed as a node, while the edge connecting two nodes represents the association between two symptoms. The R packages “qgraph” (version 1.9.4) (12) and “bootnet” (version 1.5) (57) were used to visualize the network analysis of depression, anxiety, and insomnia, where a green edge represents a positive correlation, a red edge represents a negative correlation, and a thicker edge represents a stronger correlation between nodes. The *goldbricker* function in R package networktools (58) was used to examine potential item redundancy (i.e., less than 25% of the significant different correlations). Following previous research (59–61), the item PHQ3 (“Sleep”) in the PHQ-9 was considered redundant given that another specific measure on insomnia was used, hence, PHQ3 was excluded from the network analysis.

#### 2.3.2 Centrality and stability

We estimated the centrality index of expected influence (EI) of the nodes in the network through R package “qgraph” (version 1.9.4) (12), where EI refers to the sum of the weights of the edges shared by the node symptoms and other nodes. The EI centrality was calculated by summarizing the sum of positive and negative edges connected to a specific node, which can identify the most influential nodes (i.e., symptoms) within the network model (62). A higher EI indicates a more influential the symptom in the network (62). The R package “networktools” (version 1.5.0) (58) was used to calculate the Bridge Expected Influence (BEI) index to identify bridge symptoms connecting different communities of depression, anxiety and insomnia, namely central symptoms connecting other different symptom communities (15). A higher BEI value indicates a stronger bridging effect of nodes between different communities.

In addition, the predictability index of each node was estimated using the “mgm” (version 1.2.13) (63) network model in the R package. The predictability value quantifies the extent to which a given node is likely to be explained by other neighboring nodes in the model.

The bootstrap method in R package “bootnet” (version 1.5) (57) was used to evaluate the accuracy and stability of the network model. In addition, the correlation stability coefficient (CS-coefficient) using the case-dropping bootstrap approach was used to test the centrality and stability of EI and BEI (57); values greater than 0.25 represent good stability.

#### 2.3.3 Network comparison

Previous studies (64–66) found that education levels were associated with severity of depression and anxiety; therefore, their potential influence on the network model was examined using R package “NetworkComparisonTest” (version 2.2.1) (67). Furthermore, the overall network structure, global strength, and differences at each edge between subsamples were compared between different education levels.

## 3 Results

Of the 1,564 fire service recruits who were invited to participate in this study, 1,560 met the study entry criteria and completed all the assessments. These included 479 (30.7%) recruits from Beijing, 489 (31.3%) from Sichuan, 275 (17.6%) from Guangxi and 317 (20.3%) from Guizhou, giving a participation rate of 99.7%. Of the four fire service recruits who declined to participate in this study due to lack of interest, two came from Guangxi, and one came from Beijing and Sichuan each. All participants were males. The mean age of the participants was 22.6 [standard deviation (SD): 1.8] years; namely 22.3 (SD: 1.9) years in Beijing, 22.5 (SD: 1.8) years in Sichuan, 22.7 (SD: 1.7) years in Guangxi, and 23.0 (SD: 1.8) years in Guizhou; (*p* < 0.001). A total of 874 (56.03%) had college educational and above level (44.1% in Beijing; 55.8% in Sichuan; 69.1% in Guangxi; 63.1% in Guizhou; *p* < 0.001). The prevalence of depression (PHQ-9 ≥ 5) was 15.2% (95% CI: 13.5–17.1%) (11.1% in Beijing; 20.0% in Sichuan; 13.5% in Guangxi; 15.5% in Guizhou; *p* = 0.001), while the prevalence of anxiety (GAD-7 ≥ 5) was 11.2% (95% CI: 9.6–12.8%) (6.7% in Beijing; 12.9% in Sichuan; 9.1% in Guangxi; 17.0% in Guizhou; *p* < 0.001). The overall QOL score was 3.8 (SD: 0.8) in the whole sample, while the corresponding figure was 4.0 (SD: 0.8) in Beijing; 3.7 (SD: 0.8) in Sichuan; 3.8 (SD: 0.8) in Guangxi; 3.9 (SD: 0.7) in Guizhou (*p* < 0.001).

**Figure 1:**
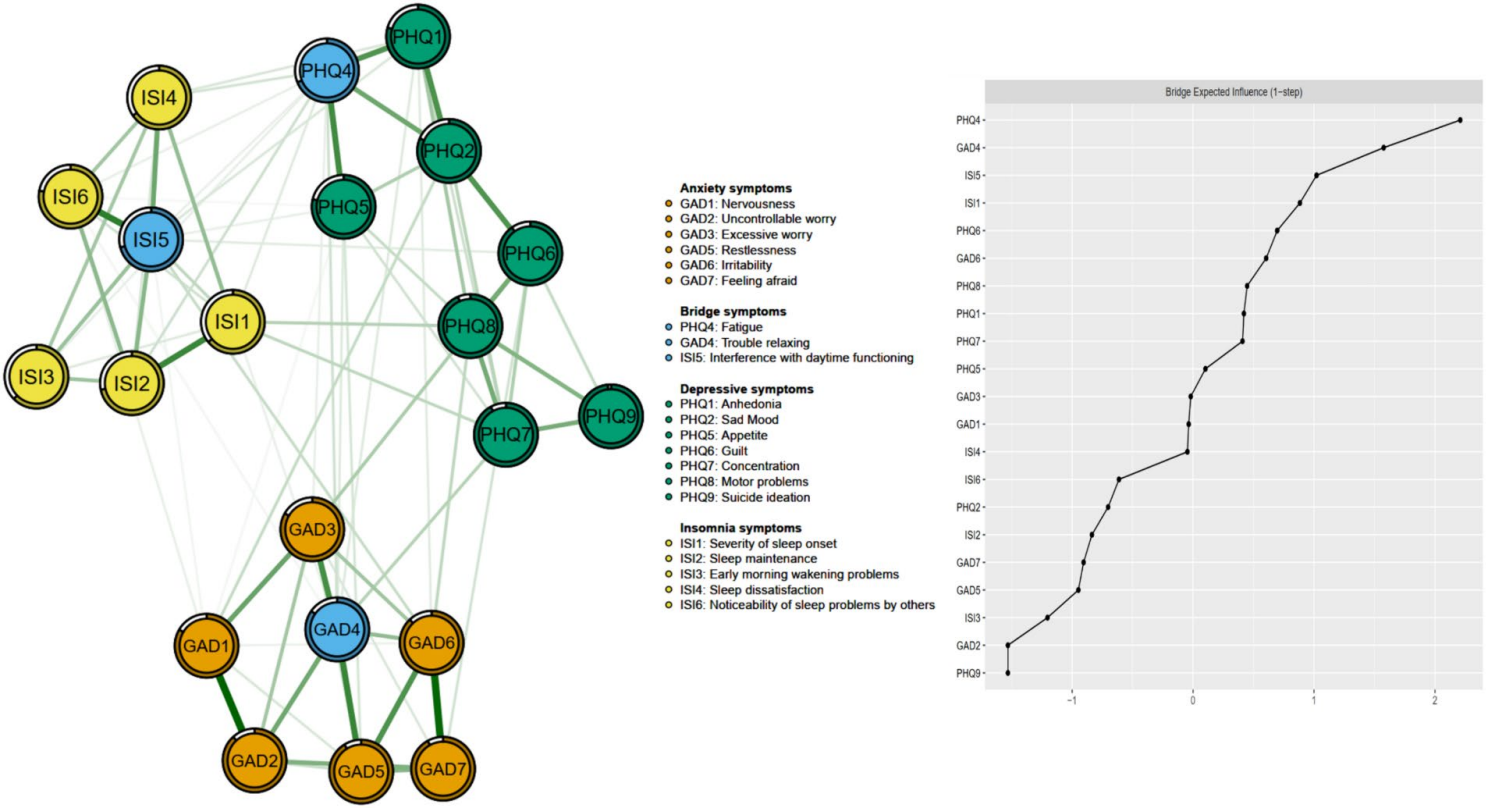
Network structure of insomnia, anxiety, and depressive symptoms showing bridge symptoms in fire service recruits during the post-COVID-19 era.

The correlation matrix of PHQ-9, GAD-7 and ISI items are shown in Supplementary Table S1. GAD1 (“Nervousness”) – GAD2 (“Uncontrollable worry”) was the strongest positive edge, followed by GAD6 (“Irritability”) – GAD7 (“Feeling afraid”) and ISI5 (“Interference with daytime functioning”) – ISI6 (“Noticeability of sleep problems by others”) (Figure 1).

### 3.1 Network structure

The item redundancy analysis did not reveal any redundant ISI or PHQ-9 items. Therefore, all items of depression, anxiety and insomnia except for PHQ3 (“Sleep”) were included for analysis.

**Figure 2:**
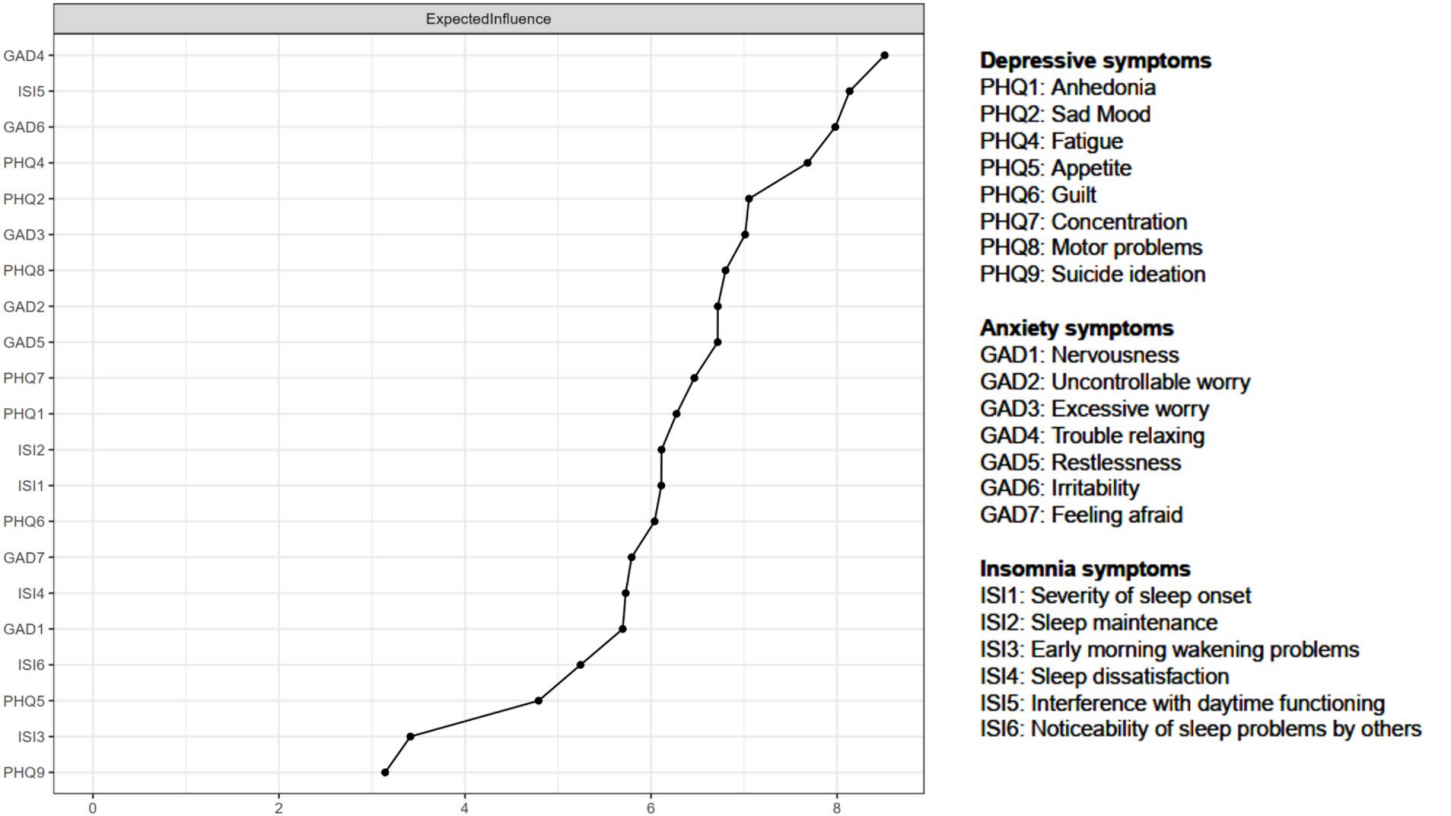
Expected influence of the network structure of depressive, anxiety and insomnia symptoms among fire service recruits during the post-COVID-19 era.

The left panel of Figure 1 shows the network structure of depression, anxiety, and insomnia symptoms. The average predictability of nodes was 0.813, ranging from 0.627 to 0.984, indicating that on average 81.3% variance of each node could be explained by its neighboring nodes in the model (Figure 1 and Table 1). Table 1 and Figure 2 shows that GAD4 “Trouble relaxing” (EI: 8.512) had the highest EI in the whole network structure, followed by ISI5 “Interference with daytime functioning” (EI: 8.135) and GAD6 “Irritability” (EI: 7.981), indicating that these were the most influential symptoms in the overall network model. In contrast, PHQ4 “Fatigue” (EI: 2.577) had the highest bridge EI values, followed by GAD4 “Trouble relaxing” (EI: 2.140) and ISI5 “Interference with daytime functioning” (EI: 1.758) in this network (Figure 1).

**Table 1:** Descriptive statistics of measurement items.

Item abbreviation	Item content	Presence^1^	Absence^2^	Expected influence^3^	Predictability
PHQ1	Anhedonia	317 (20.3%)	1,243 (79.7%)	6.274	0.796
PHQ2	Sad Mood	289 (18.5%)	1,271 (81.5%)	7.053	0.815
PHQ4	Fatigue	484 (31.0%)	1,076 (69.0%)	7.684	0.690
PHQ5	Appetite	329 (21.1%)	1,231 (78.9%)	4.791	0.789
PHQ6	Guilt	153 (9.8%)	1,407 (90.2%)	6.038	0.902
PHQ7	Concentration	124 (7.9%)	1,436 (92.1%)	6.466	0.920
PHQ8	Motor problems	99 (6.3%)	1,461 (93.7%)	6.801	0.936
PHQ9	Suicide ideation	25 (1.6%)	1,535 (98.4%)	3.142	0.984
GAD1	Nervousness	259 (16.6%)	1,301 (83.4%)	5.696	0.835
GAD2	Uncontrollable worry	181 (11.6%)	1,379 (88.4%)	6.718	0.883
GAD3	Excessive worry	259 (16.6%)	1,301 (83.4%)	7.012	0.833
GAD4	Trouble relaxing	238 (15.3%)	1,322 (84.7%)	8.512	0.847
GAD5	Restlessness	138 (8.8%)	1,422 (91.2%)	6.716	0.912
GAD6	Irritability	188 (12.1%)	1,372 (87.9%)	7.981	0.879
GAD7	Feeling afraid	126 (8.1%)	1,434 (91.9%)	5.792	0.920
ISI1	Severity of sleep onset	559 (35.8%)	1,001 (64.2%)	6.110	0.641
ISI2	Sleep maintenance	440 (28.2%)	1,120 (71.8%)	6.113	0.717
ISI3	Early morning wakening problems	582 (37.3%)	978 (62.7%)	3.413	0.627
ISI4	Sleep dissatisfaction	1,021 (65.4%)	539 (34.6%)	5.727	0.655
ISI5	Interference with daytime functioning	453 (29.0%)	1,107 (71.0%)	8.135	0.710
ISI6	Noticeability of sleep problems by others	342 (21.9%)	1,218 (78.1%)	5.240	0.780

PHQ-9, the 9-item Patient Health Questionnaire; GAD-7, 7-item Generalized Anxiety Disorder Scale; ISI, Insomnia Severity Index; SD, standard deviation; ^1^*n*(%), item recorded as 1 or 2 or 3 was considered to be “presence”; ^2^*n*(%), item recorded as 4 or 5 was considered to be “absence”; ^3^the values of expected influence were raw data generated from the network.

### 3.2 Network stability

**Figure 3:**
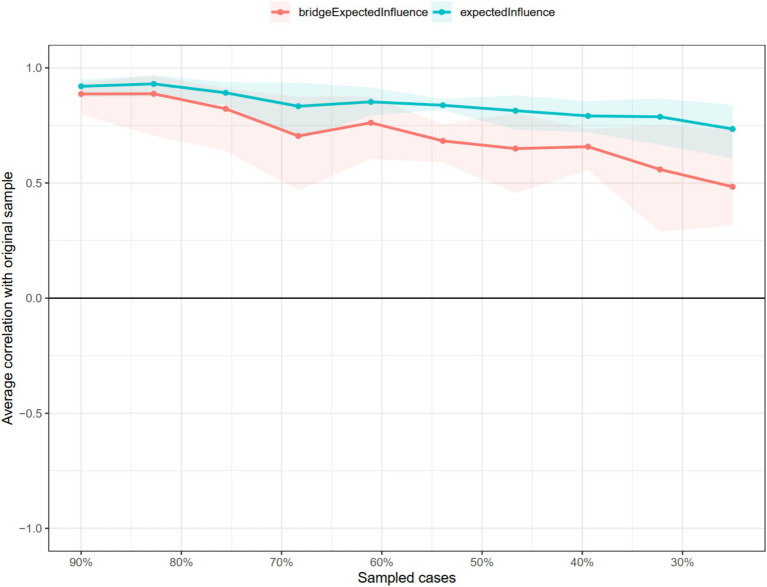
The stability of centrality and bridge centrality indices using case-dropping bootstrap.

In terms of network stability, as shown in Figure 3, the CS-coefficient of strength was 0.533, indicating excellent stability as the network structure would not significantly change even if 53.3% of the samples were removed. In addition, the CS-coefficient of the bridge strength obtained by the case dropping bootstrap method was 0.244 (Figure 3). The edge-weight bootstrap difference test showed that most comparisons between edge weights were statistically significant (Supplementary Figure S1). Moreover, the bootstrapped stability test for EI showed that these central symptom nodes were statistically stronger than other nodes, indicating that the primary results were reliable (Supplementary Figure S2). Therefore, the network model demonstrated acceptable reliability and stability.

### 3.3 Flow network of quality of life

**Figure 4:**
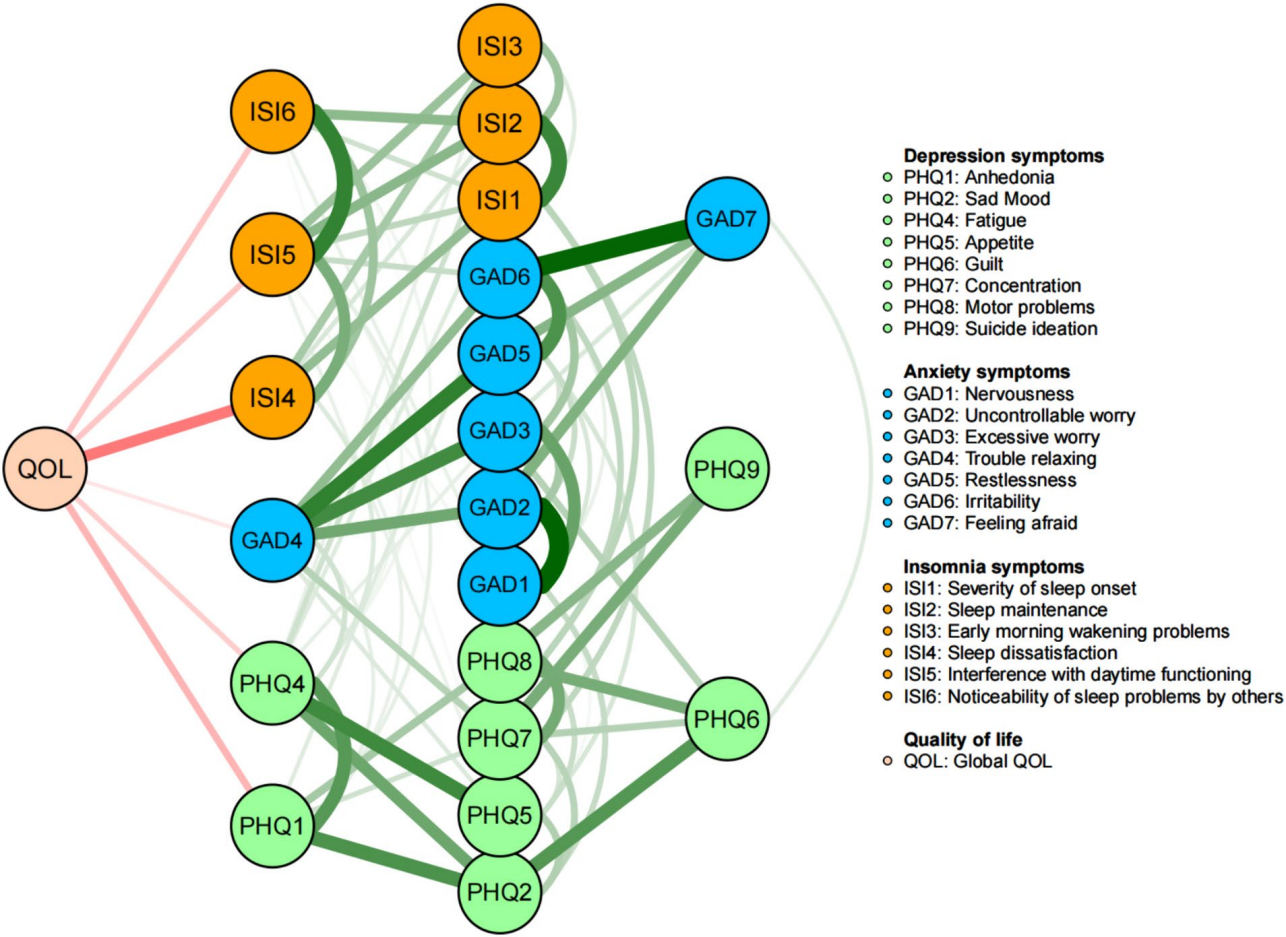
Flow network of quality of life.

Figure 4 shows the flow network model of PHQ-9, GAD-7 and ISI symptoms and QOL. ISI4 “Sleep dissatisfaction” (average edge weight = −1.335), as the central symptom with the highest intensity value, had the strongest negative correlation with QOL, followed by PHQ1 “Anhedonia” (average edge weight = −0.741) and ISI6 “Noticeability of sleep problems by others” (average edge weight = −0.589).

### 3.4 Network comparison test by educational level (high school and lower vs. above high school)

Network models by educational level are shown in Supplementary Figure S3. In the comparison of different models, no significant difference was found in the network global strengths (high school and lower: 58.357; above high school: 57.694; S = 0.662, *p* = 0.97; Supplementary Figure S3). There was also no significant difference in the network structure-distribution of edge weights (*M* = 1.640, *p* = 0.59).

## 4 Discussion

This study was the first network analysis to investigate the interrelationships between depression, anxiety and insomnia symptoms among Chinese fire service recruits in the post-COVID-19 era. The prevalence of depression and anxiety in this sample was 15.2% (95% CI: 13.5–17.1%) and 11.2% (95% CI: 9.6–12.8%), respectively, which are lower than the corresponding prevalence of depression (45.3%) and anxiety (48.1%) among medical trainees in New York (68), and that of depression (22.8%; 95% CI: 20.22–25.32) and anxiety (20.0%; 95% CI: 17.55–22.41) among the general population in Ireland during the pandemic (69). However, our findings are higher than the prevalence of depression (8.4%; PHQ-9 ≥ 10) and anxiety (4.8%; GAD-7 ≥ 10) among the general population aged 35–69 in Russian in the pre-COVID-19 period (70). The possible reasons for the discrepancy included differences in the study samples, sampling methods, assessment tools on depression and anxiety, and stages of the pandemic. For instance, a study found that compared with the figures at the peak of the COVID-19 pandemic, the prevalence of depression and anxiety among participants increased after the COVID-19 peak (71). Further, the long-term effects of COVID-19 might also perpetuate or exacerbate mental health problems (72). Although daily physical training could reduce the risk and severity of depression and anxiety (73, 74), other factors could offset the benefits. These include long-term heavy physical training, strict isolation measures, fear of infectious transmission, personal uncertainty about future career development, and lack of social and family support, all of which could increase risk of depression, anxiety and sleep disturbances among fire service recruits during and immediately after the COVID-19 pandemic, (75, 76).

We found that differences in prevalence of depression and anxiety across the four provinces in China, with Beijing having the lowest prevalence and Sichuan having the highest prevalence. Possible reasons included the differences in the stages of the COVID-19 pandemic wave, prevention and control policies, and economic conditions between provinces (77, 78). Compared with most of other areas in China, Beijing, as the capital of China, had stricter public health policies, better economic conditions, and better social support (79, 80), all of which were associated with lower risk of depression and anxiety than that of other provinces (77).

In this network analysis, GAD4 “Trouble relaxing” was a central and bridge symptom, and therefore one of the most influential symptom in maintaining the whole network model, which is consistent with previous findings reported in Macau residents, Filipino domestic helpers in Macau, and Chinese female nursing students (21, 81, 82). Interventions that target this symptom might be effective in reducing the severity of related symptoms in the anxiety-depression-sleep network (14). A study of psychopathological symptom network in the COVID-19 outbreak and post-peak periods found that “inability to relax” symptoms played an important role with high centrality during the COVID-19 outbreak, but significantly reduced after the COVID-19 peak (71). For the Chinese fire service recruits in the post-COVID-19 era, the long-term closed training coupled with the strict lockdown measures likely resulted in marked exhaustion and a lack of ability to rest or relax (83). In addition, such quarantine reduced communication with the outside world as well as entertainment activities that served as coping mechanisms for long-term state of tension (84), thus increasing the risk for anxiety symptoms and other mental health problems among fire service recruits.

GAD6 “Irritability,” which refers to a low threshold for anger due to frustration (85, 86), is a diagnostic criterion for generalized anxiety disorder (87). “Irritability” was a central symptom in this study, which is consistent with previous findings among adolescents (17). According to the relevant regulations and policies of China’s National comprehensive fire protection, fire service recruits need to undergo arduous physical training and professional assessments in order to meet the required standards to qualify as firefighters (1). The fear of failing such strict assessments and meeting the high standards could result in irritability and anxiety among the fire service recruits.

Another central and bridge symptom identified in this study was ISI5 (“Interference with daytime functioning”), which might exacerbate symptoms of depression, anxiety, and insomnia (19, 21). This is consistent with another network analysis study conducted among clinicians in China, in which “Interference with daytime functioning” was the most influential symptom in the network model of anxiety, insomnia, and depressive symptoms (59). Previous studies found that physical discomfort and fatigue are significantly associated with daytime functioning caused by sleep disturbances (88, 89). The rigorous and continuous high-intensity physical training could lead to extreme physical fatigue, susceptibility to sports injuries, and increased risk of insomnia, all of which could interfere with their normal daytime function. Therefore, exploring ways to address inability to relax, irritability, and daytime functioning might reduce the risk of their mental health problems in fire service recruits during the post-pandemic era.

Fatigue is a common health issue across many populations; for instance, the prevalence of fatigue was found to affect up to 83.5% of nursing students (90). In this study, PHQ4 (“Fatigue”) was both the central and bridge symptom in the depression-anxiety-insomnia network, which supports previous network analysis findings among clinicians working in public hospitals (59). In a previous network analysis study of depressive and anxiety symptoms among female nursing students, “fatigue” was also the most central symptom (82). Similar results were also reported in another network study involving residents in Guangdong province of China (91). Fatigue and insomnia symptoms often co-occur in patients with depression, and those with fatigue usually have a higher risk of depression (92). Moreover, excessive activities and insomnia could increase the risk of fatigue (90). After ceasing the dynamic zero-COVID policy in China, fire service recruits remained prone to experience physical fatigue and depression (93), which might be partly due to ongoing rigorous physical training, strict isolation and a lack social support and recreational activities. Therefore, the occurrence and development of mental health problems, including physical fatigue and negative emotions persisted (94).

In network theory, bridge symptoms are considered transdiagnostic, which means that interventions targeting bridge symptoms, such as “fatigue,” could be effective for all the three communities in this study, i.e., depression, anxiety and insomnia (59, 95). Previous research found that individuals who reported fatigue had a higher risk of depression, and interventions targeting fatigue could relieve depressive and anxiety symptoms (91). Therefore, improving fatigue might influence the severity and dynamics of the communities of depression, anxiety and insomnia network. For example, cognitive behavioral therapy, practical rehabilitation treatment, supportive listening counseling interventions and self-guided online interventions appeared to be effective and accessible measures for fatigue in primary care (96, 97). Moreover, traditional Chinese medicine (TCM) treatment such as acupuncture and moxibustion could also improve fatigue symptoms (98, 99), through regulating immune dysfunction and abnormal activities of the hypothalamic–pituitary–adrenal (HPA) axis (100). These interventions for fatigue might be effective in reducing the severity and interaction of depression, anxiety and insomnia symptoms in patients (101).

The QOL is a multidimensional and significant health outcome indicator of an individual’s overall well-being, encompassing both physical and mental health (102). Prior studies demonstrated the negative relationship between QOL and various mental health problems such as insomnia, depressive (specifically anhedonia) and anxiety symptoms and suicidal ideation across different populations (82, 103–107). As expected, ISI4 “Sleep dissatisfaction” had the strongest negative correlation with QOL among fire service recruits in this study, which is consistent with previous findings among Macau residents (21). Hence, reducing the occurrence of insomnia symptoms, such as “Sleep dissatisfaction,” might play an important role in improving QOL. Insomnia could lead to a variety of physical and mental health problems, including cardiovascular disease, depression, anxiety, and suicide (108–110), which could affect daytime function, normal work and life activities, and QOL (111). During the COVID-19 pandemic, the rigorous physical training in an isolated environment (112), could have affected sleep satisfaction and lowered QOL among new fire service recruits in China.

PHQ1 (“Anhedonia”) was another symptom that was negatively associated with QOL, indicating the influential role of anhedonia on QOL among fire service recruits. For fire service recruits, the fear of COVID-19 infection, isolation from their families/friends due to the nature of their work (113), and the uncertainty of the future (114), might have aggravated the concerns about their future career development and life (115, 116). These factors could increase the risk of depressive symptoms including anhedonia, and thereby reducing their QOL (117–119).

The strengths of this study consisted of the inclusion of an under-studied sample, a large sample size and the use of novel and sophisticated statistical analyses. However, several limitations should be acknowledged. First, due to the cross-sectional nature of the study, it was not possible to assess dynamic changes of individual symptoms or establish causal relationships between the symptoms. Second, due to the restrictive measures during the COVID-19 pandemic, this study relied solely on online self-report to assess various symptoms, which might have resulted in recall and reporting bias. Third, for logistical reasons, diagnostic instruments on psychiatric disorders were not used. Therefore, the diagnoses of anxiety and depressive disorders among fire service recruits could not be examined.

In conclusion, depression and anxiety were key mental health problems to address among fire service recruits in the post-COVID-19 era. ISI4 “Sleep dissatisfaction” was the most central symptom, with the strongest negative correlation with QOL, followed by PHQ1 “Anhedonia” and ISI6 “Noticeability of sleep problems by others.” In addition, the central and bridge symptoms (e.g., GAD4, ISI5, GAD6, and PHQ4) in this network analysis warrant attention when addressing depression and anxiety and developing future targeted interventions in this population.

## Data availability statement

The datasets presented in this article are not readily available because the the Ethics Committee of the Emergency General Hospital that approved the study prohibits the authors from making publicly available the research dataset of clinical studies. Requests to access the datasets should be directed to xyutly@gmail.com.

## Ethics statement

The study was approved by the ethics committee of the Emergency General Hospital and all participants signed the electronic written informed consent.

## Author contributions

JL: Data curation, Investigation, Writing – review & editing. ZG: Conceptualization, Data curation, Formal analysis, Investigation, Methodology, Project administration, Software, Writing – original draft, Writing – review & editing. PC: Data curation, Formal analysis, Writing – review & editing, Methodology. HC: Writing – review & editing, Data curation, Formal analysis. YF: Data curation, Writing – review & editing. T-IH: Data curation, Writing – review & editing. S-YR: Data curation, Writing – review & editing. ZS: Data curation, Writing – review & editing. TC: Data curation, Writing – review & editing. CN: Writing – review & editing. GW: Data curation, Writing – review & editing, Funding acquisition. Y-TX: Data curation, Writing – review & editing, Formal analysis, Investigation, Methodology, Project administration, Resources, Software, Writing – original draft.
