# Supplementary material for: A network analysis of the interrelationships between depression, anxiety, insomnia and quality of life among fire service recruits
Source: Front Public Health. 2024 Jul 3;12:1348870. doi: 10.3389/fpubh.2024.1348870 (PMC11252005; doi:10.3389/fpubh.2024.1348870)
Supplement: Supplementary file 1 [file Data_Sheet_1.docx]

**Supplementary Materials**

Figure S1. Estimation of edge difference within the depression-anxiety-insomnia network by bootstrapped difference test

Figure S2.Estimation of node expected difference within the depression-anxiety-insomnia network by bootstrapped difference test

Figure S3. Network comparison of insomnia, anxiety, depressive symptoms between educational levels

Table S1 Correlation matrix of PHQ-9, GAD-7 and ISI items

|  | PHQ1 | PHQ2 | PHQ4 | PHQ5 | PHQ6 | PHQ7 | PHQ8 | PHQ9 | GAD1 | GAD2 | GAD3 | GAD4 | GAD5 | GAD6 | GAD7 | ISI1 | ISI2 | ISI3 | ISI4 | ISI5 | ISI6 |
| --- | --- | --- | --- | --- | --- | --- | --- | --- | --- | --- | --- | --- | --- | --- | --- | --- | --- | --- | --- | --- | --- |
| PHQ1 | 0.000 | 1.801 | 1.748 | 0.000 | 0.000 | 0.481 | 0.899 | 0.000 | 0.000 | 0.000 | 0.000 | 0.306 | 0.000 | 0.293 | 0.000 | 0.000 | 0.000 | 0.000 | 0.378 | 0.365 | 0.000 |
| PHQ2 | 1.801 | 0.000 | 1.453 | 0.777 | 1.749 | 0.701 | 0.000 | 0.000 | 0.570 | 0.000 | 0.000 | 0.000 | 0.000 | 0.000 | 0.000 | 0.000 | 0.000 | 0.000 | 0.000 | 0.000 | 0.000 |
| PHQ4 | 1.748 | 1.453 | 0.000 | 1.905 | 0.000 | 0.000 | 0.000 | 0.000 | 0.000 | 0.000 | 0.224 | 0.475 | 0.000 | 0.000 | 0.000 | 0.300 | 0.478 | 0.225 | 0.430 | 0.217 | 0.223 |
| PHQ5 | 0.000 | 0.777 | 1.905 | 0.000 | 0.000 | 0.432 | 0.551 | 0.000 | 0.105 | 0.000 | 0.000 | 0.329 | 0.401 | 0.000 | 0.000 | 0.000 | 0.000 | 0.000 | 0.000 | 0.211 | 0.077 |
| PHQ6 | 0.000 | 1.749 | 0.000 | 0.000 | 0.000 | 0.789 | 1.384 | 0.580 | 0.000 | 0.000 | 0.816 | 0.000 | 0.000 | 0.000 | 0.431 | 0.000 | 0.000 | 0.000 | 0.000 | 0.285 | 0.000 |
| PHQ7 | 0.481 | 0.701 | 0.000 | 0.432 | 0.789 | 0.000 | 1.384 | 1.341 | 0.000 | 0.000 | 0.000 | 0.703 | 0.000 | 0.000 | 0.000 | 0.633 | 0.000 | 0.000 | 0.000 | 0.000 | 0.000 |
| PHQ8 | 0.899 | 0.000 | 0.000 | 0.551 | 1.384 | 1.384 | 0.000 | 1.219 | 0.000 | 0.000 | 0.000 | 0.000 | 0.000 | 0.633 | 0.000 | 0.729 | 0.000 | 0.000 | 0.000 | 0.000 | 0.000 |
| PHQ9 | 0.000 | 0.000 | 0.000 | 0.000 | 0.580 | 1.341 | 1.219 | 0.000 | 0.000 | 0.000 | 0.000 | 0.000 | 0.000 | 0.000 | 0.000 | 0.000 | 0.000 | 0.000 | 0.000 | 0.000 | 0.000 |
| GAD1 | 0.000 | 0.570 | 0.000 | 0.105 | 0.000 | 0.000 | 0.000 | 0.000 | 0.000 | 2.577 | 1.502 | 0.000 | 0.380 | 0.206 | 0.000 | 0.000 | 0.000 | 0.000 | 0.000 | 0.134 | 0.219 |
| GAD2 | 0.000 | 0.000 | 0.000 | 0.000 | 0.000 | 0.000 | 0.000 | 0.000 | 2.577 | 0.000 | 0.894 | 1.426 | 0.540 | 0.000 | 1.278 | 0.000 | 0.000 | 0.000 | 0.000 | 0.000 | 0.000 |
| GAD3 | 0.000 | 0.000 | 0.224 | 0.000 | 0.816 | 0.000 | 0.000 | 0.000 | 1.502 | 0.894 | 0.000 | 1.863 | 0.422 | 0.920 | 0.367 | 0.000 | 0.000 | 0.000 | 0.000 | 0.000 | 0.000 |
| GAD4 | 0.306 | 0.000 | 0.475 | 0.329 | 0.000 | 0.703 | 0.000 | 0.000 | 0.000 | 1.426 | 1.863 | 0.000 | 1.996 | 1.084 | 0.000 | 0.000 | 0.000 | 0.000 | 0.213 | 0.000 | 0.111 |
| GAD5 | 0.000 | 0.000 | 0.000 | 0.401 | 0.000 | 0.000 | 0.000 | 0.000 | 0.380 | 0.540 | 0.422 | 1.996 | 0.000 | 1.779 | 1.195 | 0.000 | 0.000 | 0.000 | 0.000 | 0.000 | 0.000 |
| GAD6 | 0.293 | 0.000 | 0.000 | 0.000 | 0.000 | 0.000 | 0.633 | 0.000 | 0.206 | 0.000 | 0.920 | 1.084 | 1.779 | 0.000 | 2.519 | 0.000 | 0.000 | 0.000 | 0.000 | 0.543 | 0.000 |
| GAD7 | 0.000 | 0.000 | 0.000 | 0.000 | 0.431 | 0.000 | 0.000 | 0.000 | 0.000 | 1.278 | 0.367 | 0.000 | 1.195 | 2.519 | 0.000 | 0.000 | 0.000 | 0.000 | 0.000 | 0.000 | 0.000 |
| ISI1 | 0.000 | 0.000 | 0.300 | 0.000 | 0.000 | 0.633 | 0.729 | 0.000 | 0.000 | 0.000 | 0.000 | 0.000 | 0.000 | 0.000 | 0.000 | 0.000 | 1.972 | 0.316 | 1.063 | 0.638 | 0.455 |
| ISI2 | 0.000 | 0.000 | 0.478 | 0.000 | 0.000 | 0.000 | 0.000 | 0.000 | 0.000 | 0.000 | 0.000 | 0.000 | 0.000 | 0.000 | 0.000 | 1.972 | 0.000 | 0.977 | 0.377 | 1.172 | 1.133 |
| ISI3 | 0.000 | 0.000 | 0.225 | 0.000 | 0.000 | 0.000 | 0.000 | 0.000 | 0.000 | 0.000 | 0.000 | 0.000 | 0.000 | 0.000 | 0.000 | 0.316 | 0.977 | 0.000 | 0.897 | 0.995 | 0.000 |
| ISI4 | 0.378 | 0.000 | 0.430 | 0.000 | 0.000 | 0.000 | 0.000 | 0.000 | 0.000 | 0.000 | 0.000 | 0.213 | 0.000 | 0.000 | 0.000 | 1.063 | 0.377 | 0.897 | 0.000 | 1.458 | 0.908 |
| ISI5 | 0.365 | 0.000 | 0.217 | 0.211 | 0.285 | 0.000 | 0.000 | 0.000 | 0.134 | 0.000 | 0.000 | 0.000 | 0.000 | 0.543 | 0.000 | 0.638 | 1.172 | 0.995 | 1.458 | 0.000 | 2.112 |
| ISI6 | 0.000 | 0.000 | 0.223 | 0.077 | 0.000 | 0.000 | 0.000 | 0.000 | 0.219 | 0.000 | 0.000 | 0.111 | 0.000 | 0.000 | 0.000 | 0.455 | 1.133 | 0.000 | 0.908 | 2.112 | 0.000 |

Note: PHQ1- Anhedonia, PHQ2-Sad mood, PHQ4-Fatigue, PHQ5- Appetite, PHQ6- Worthless, PHQ7-Concentration, PHQ8-Motor, PHQ9-Death;

GAD1- Nervous, GAD2-Uncontrollable worry, GAD3- Excessive worry, GAD4- Trouble relaxing, GAD5- Restlessness, GAD6- Irritability, GAD7- Feeling afraid;

ISI1- Severity of sleep onset, ISI2- Sleep maintenance, ISI3- Early morning wakening problems, ISI4- Sleep dissatisfaction, ISI5- Interference with daytime functioning, ISI6- Noticeability of sleep problems by others;


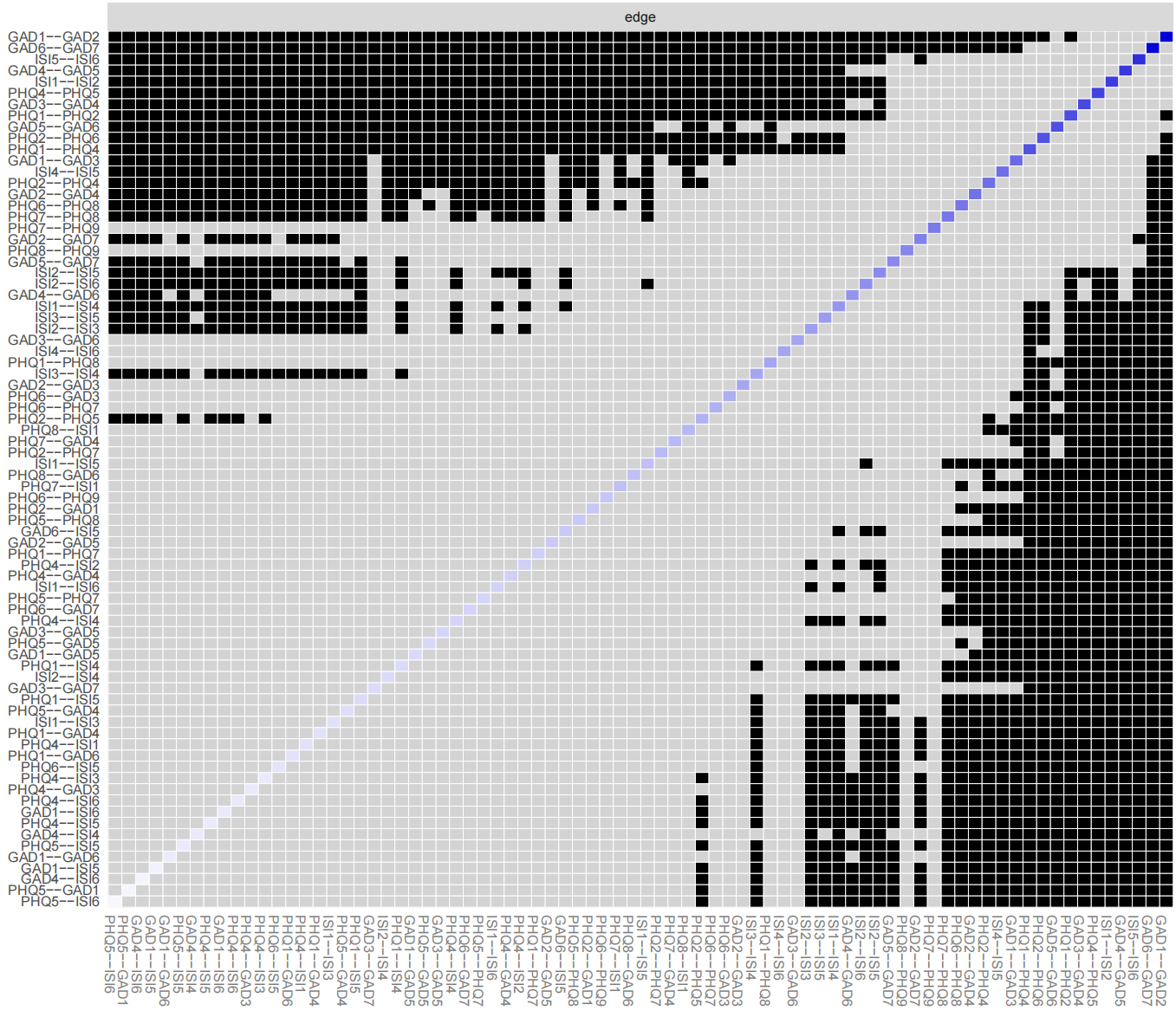


Figure S1. Estimation of edge difference within the depression-anxiety-insomnia network by bootstrapped difference test


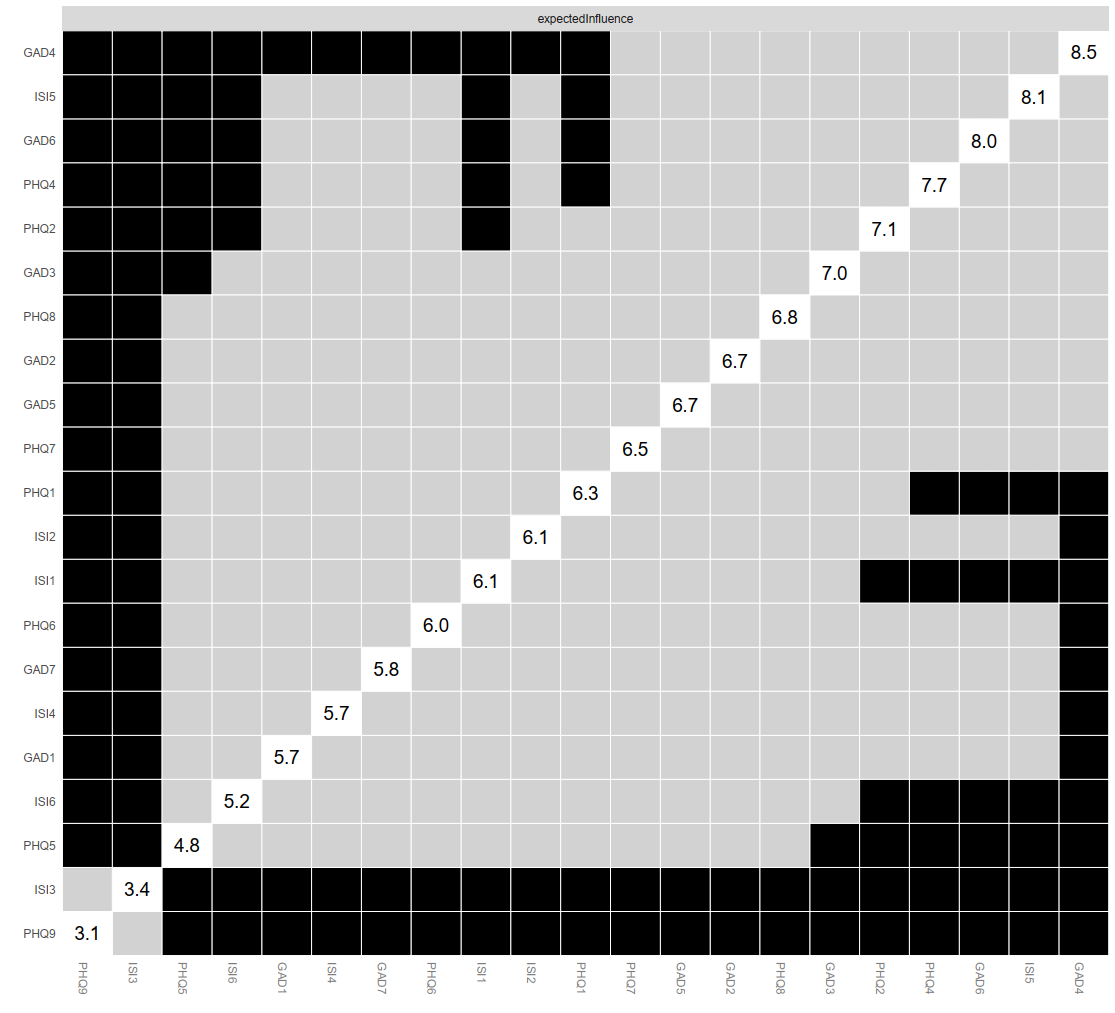


Figure S2. Estimation of node expected difference within the depression-anxiety-insomnia network by bootstrapped difference test


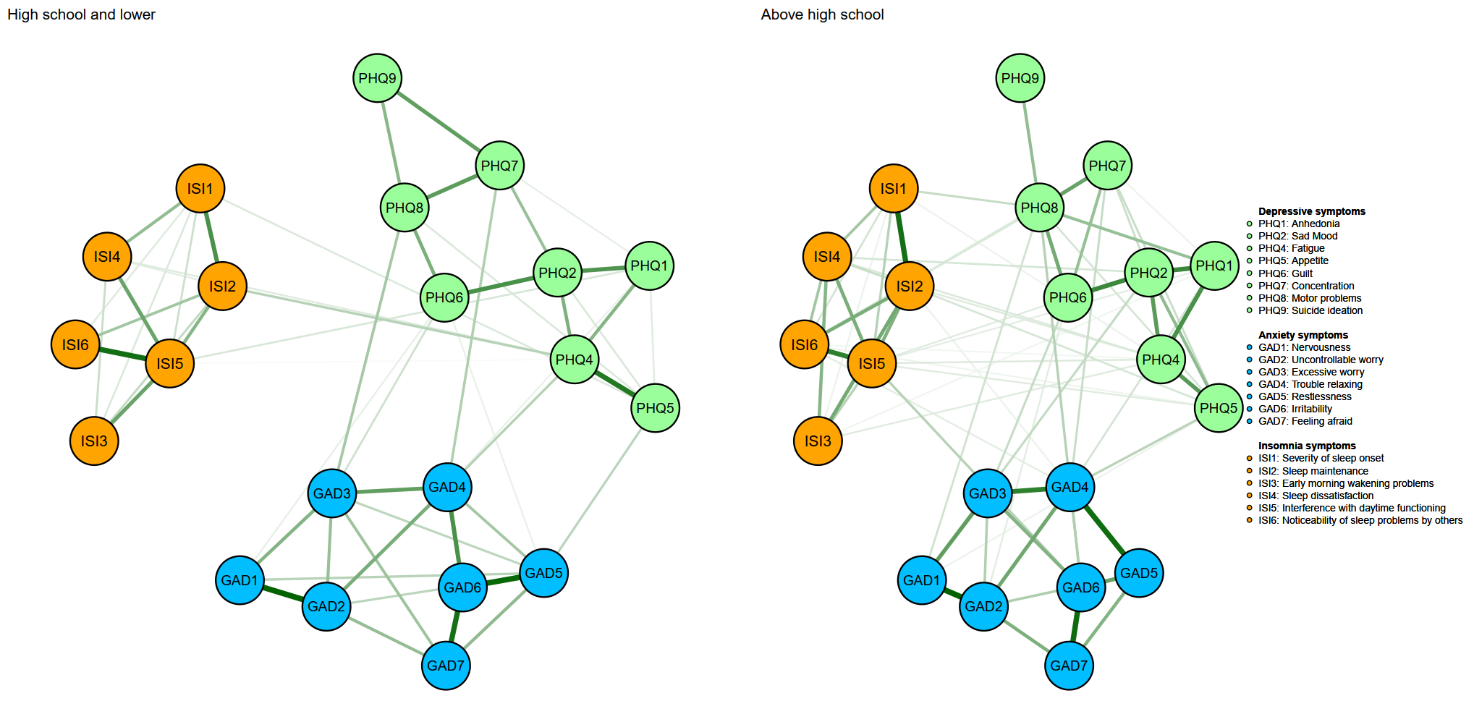


Figure S3. Network comparison of insomnia, anxiety, depressive symptoms between different educational levels
